# SARS-CoV-2 reinfection: a case report from Portugal

**DOI:** 10.1590/0037-8682-0002-2021

**Published:** 2021-03-22

**Authors:** Ana Carolina Fernandes, Rui Figueiredo

**Affiliations:** 1 Administração Regional de Saúde de Lisboa e Vale do Tejo, Lisboa, Portugal.; 2 Centro Hospitalar do Médio Tejo, Serviço de Patologia Clínica, Tomar, Portugal.

Dear Editor,

In Europe, reinfection by severe acute respiratory syndrome coronavirus 2 (SARS-CoV-2) has rarely been reported, but it is appearing worldwide^1^ ^,^ ^2^. The coronavirus disease 2019 (COVID‐19) pandemic began in December 2019 in Wuhan, China. Since then, it has rapidly spread across the globe and impacted several countries^3^. The World Health Organization (WHO) declared COVID-19 a pandemic. Reverse transcriptase-polymerase chain reaction (RT-PCR)-based assays are currently used as reference diagnostic tests. A positive result does not confirm the presence of infection, and viral RNA shedding declines after the resolution of symptoms. The maximum shedding duration is reported to be 83 days in the upper respiratory tract, 59 days in the lower respiratory tract, 126 days in the stool, and 60 days in the serum^4^.

Here, we report the case of 28-year-old asthmatic man diagnosed with COVID-19 on the 27^th^ of March 2020 in the city of Santarém (Portugal). The first symptoms of low fever, chills, and sneezing started 1 week before arrival in Barcelona (Spain). A positive result on RT-PCR assay confirmed the presence of SARS-CoV-2, and the patient was isolated at home. He was prescribed an antipyretic, antihistaminic, and expectorant. On April 24^th^ and again on April 27^th^ two negative RT-PCR results were obtained, and the patient was assumed to be cured. Eight months later, on 14^th^ December, a routine rapid antigen test result was negative. On 17^th^ December, after close contact with a COVID-19-positive colleague, he again showed symptoms of COVID-19 infection. This time, the symptoms were more severe than those in the previous episode, with fever, tiredness, productive cough, frontal headache, dizziness, dark urine, and dysuria, but no anosmia and ageusia. The RT-PCR test results were positive on the 19^th^ of December for SARS-CoV-2. His pulse was 102/min during rest with oxygen saturation of 97%. Cardiopulmonary auscultation showed no changes.

At least 111 million people worldwide are infected with SARS-CoV-2 that has caused almost 2.5 million deaths^5^. One year after the first case of COVID-19 was reported, not much is known about reinfection with SARS-CoV-2 although the scientific community around the world started finding strong evidence for it. A rigorous genetic study is needed to provide evidence of a distinct SARS-CoV-2 strain in the second infection. Reinfection by SARS-CoV-2 in a human was confirmed for the first time in August 2020 in Hong Kong, with a difference of 142 days^6^. In our case report, the interval between the first infection and reinfection was 8 months (236 days). Our patient who was infected in Barcelona and later by contact with a colleague was thought to be infected by two different strains of SARS-CoV-2. To the best of our knowledge, this is the first report of reinfection in Portugal. The immune response of the patient may have decreased or was not adapted to mutations of SARS-CoV-2, which has a rate of two mutations per month^7^. It is presumed that people will become vulnerable to COVID-19 again after recovering from a prior infection, depending on how the immune system typically responds to other respiratory viruses, including other coronaviruses^8^. Therefore, this case report presents strong evidence that SARS-CoV-2 reinfection, although rare, is accompanied by severe symptoms.
